# Neuron-Astrocyte Interactions and Circadian Time-keeping in Mammals

**DOI:** 10.1177/10738584241245307

**Published:** 2024-04-11

**Authors:** Nicola J. Smyllie, Michael H. Hastings, Andrew P. Patton

**Affiliations:** Medical Research Council Laboratory of Molecular Biology, Cambridge, U.K

**Keywords:** Neuropeptides, glutamate, GABA, retina, Period, Cryptochrome, biological clock, suprachiasmatic nucleus, hypothalamus, sleep

## Abstract

Almost every facet of our behaviour and physiology varies predictably over the course of day and night, anticipating, and adapting us to, their associated opportunities and challenges. These rhythms are driven by endogenous biological clocks that, when deprived of environmental cues, can continue to oscillate with a period of approximately one day, hence *circa*-*dian*. Normally, retinal signals synchronise them to the cycle of light and darkness, but disruption of circadian organisation, a common feature of modern lifestyles, carries considerable costs to health. Circadian time-keeping pivots around a cell-autonomous molecular clock, widely expressed across tissues. These cellular timers are in turn synchronised by the principal circadian clock of the brain, the hypothalamic suprachiasmatic nucleus (SCN). Intercellular signals make the SCN network a very powerful pacemaker. Previously, neurons were considered the sole SCN timekeepers, with glial cells playing supportive roles. New discoveries have revealed, however, that astrocytes are active partners in SCN network timekeeping, their cell-autonomous clock regulating extracellular glutamate and GABA concentrations to control circadian cycles of SCN neuronal activity. Here, we introduce circadian timekeeping at the cellular and SCN network levels before focussing on the contributions of astrocytes and their mutual interaction with neurons in circadian control in the brain.

## Introduction

Daily rhythms of physiology and behaviour pervade biology, adapting organisms to regular changes to their environment. These rhythms are driven by powerful endogenous timing mechanisms, “biological clocks” that maintain their autonomous oscillation with a period of approximately one day (hence, *circa*-*dian*) in the absence of environmental cues. Under natural conditions, however, circadian clocks are synchronised by the light-dark cycle and become predictive of solar time, preparing organisms for the alternating demands of day and night. In mammals, the cycle of sleep and wakefulness is the most obvious circadian rhythm, and through this, the circadian timing system controls almost every aspect of brain function. Moreover, it can do so independently of its immediate control over the sleep/wake cycle itself, with circadian rhythms of cognition, mood, alertness and brain homeostasis persisting even when experimental subjects are deprived of sleep (Czeisler and Gooley 2007). Beyond the brain, the circadian timing system drives oscillations of cellular and tissue-level metabolism, thereby ensuring that daily rhythms of physiology and behaviour are co-ordinately matched across the organism (Reppert and Weaver 2002). A fully functional circadian system is therefore integral for our health and wellbeing. Indeed, numerous studies have linked disrupted circadian time-keeping with sleep disorders (Toh and others 2001), psychiatric disease (Karatsoreos 2014) and neurological conditions (Colwell 2021): it appears that a disrupted clock and disease progression are intimately, and reciprocally, entwined.

The principal circadian clock in mammals is the suprachiasmatic nucleus (SCN) of the hypothalamus: a network that consists of ~10,000 neurons and ~3,000 glia flanking the third ventricle, immediately above the optic chiasm and receiving retinal input via the retinohypothalamic tract (RHT) [Box 1]. It generates its own daily cycle of neuronal activity, which peaks in circadian daytime and persists in the absence of environmental input. The daily cues encoded by this cycle of neuronal firing impose temporal coherence across tissues and thereby ensure metabolic efficiency within the organism. Given its small size, the SCN is a remarkable piece of neural circuitry because it sets the tempo to our life: but how does it achieve this? Cell-autonomous circadian time-keeping is the starting point, but it is now clear that interactions across its neuronal network confer the uniquely defining properties of the SCN clock: robust, high-amplitude oscillation with a precisely defined and stable period. Interneuronal signalling, mediated over long (hourly) time-scales by paracrine neuropeptidergic cues, is critical for this close synchronisation, and until recently, it was thought that neurons alone were responsible for encoding time within the SCN. A growing body of evidence, however, now highlights a significant role for glia, particularly astrocytes, in the SCN time-piece. Not only do astrocytes have a cell-autonomous clock comparable to that of neurons, but SCN astrocytes can drive circadian behaviour in the absence of any neuronal clocks (Brancaccio and others 2019). In this review, we introduce the molecular mechanisms that are at the core of the mammalian cell-autonomous clock, before focussing on the neurons and neural networks of the SCN. We then explore the contribution of the cell-autonomous clock of astrocytes and their relationship with neurons, to the control of circadian time-keeping in the SCN and more broadly across the brain.

### The molecular and cellular basis of circadian timekeeping in mammals

**Figure 1:**
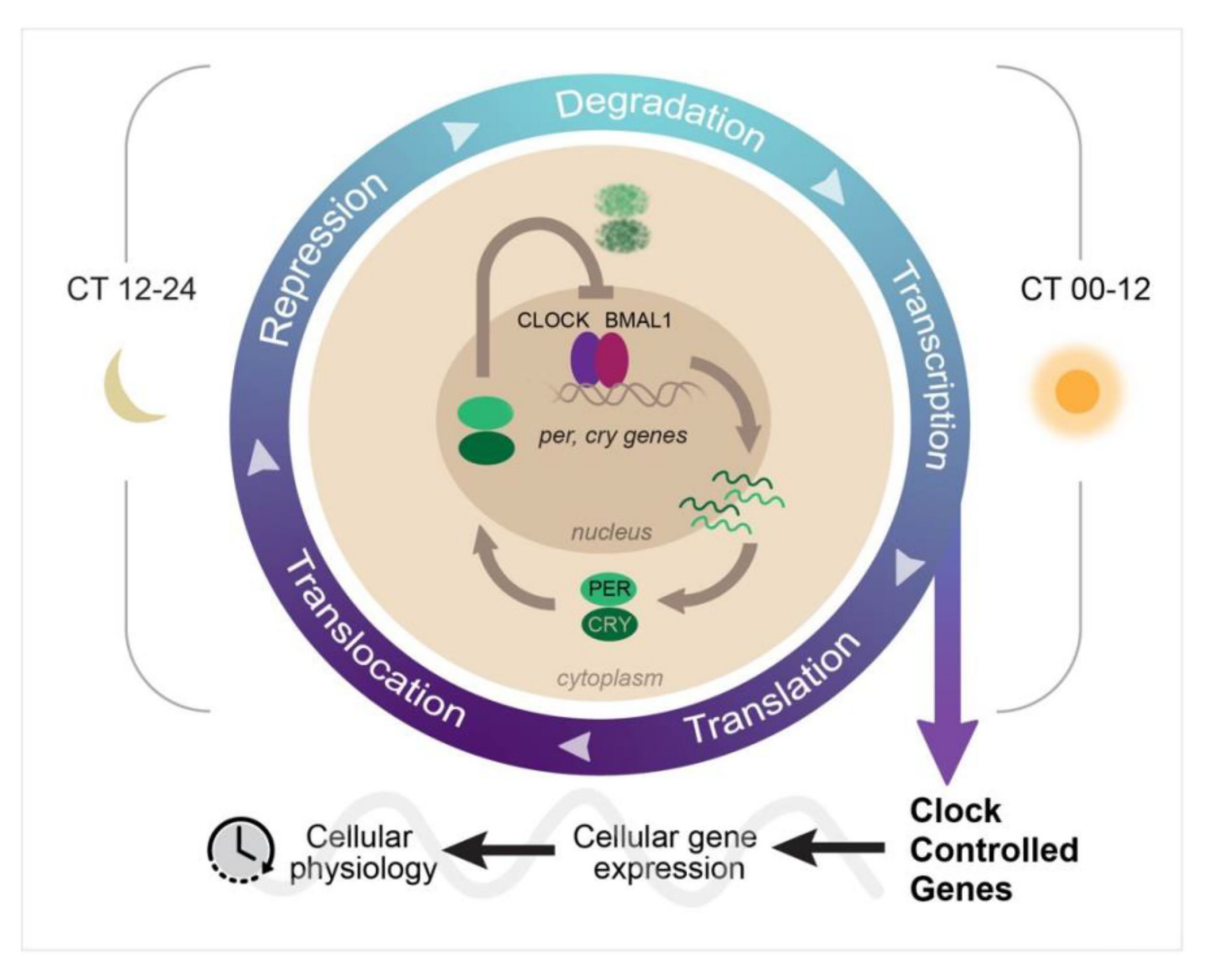
The core circadian transcriptional-translational feedback loop. Schematic of the core circadian transcriptional-translational feedback loop (TTFL) focussing on the interplay between CLOCK/ BMAL1 positive and PER/ CRY negative regulators, as shown in the centre. The TTFL progresses through stages of transcription, translation, translocation, repression and degradation, as indicated on the outer track, as PER and CRY proteins are synthesised and then degraded on a daily basis. The TTFL also directs the temporal control of transcription of clock-controlled genes that ultimately control programmes of cellular physiology. Circadian time (phase) is shown either side of the schematic: circadian day (right) and night (left).

Our understanding of the molecular-genetic basis of the clock of mammals came through comparative studies of the clock of *Drosophila* (Konopka and Benzer 1971), and by mutagenesis screens and targeted gene-editing in mice (King and others 1997). Further biochemical and cellular validation in mouse and human has demonstrated the general applicability of a model that has, at its core, a negative transcriptional/ post-translational feedback loop (TTFL). This progresses through interlocked stages of transcription, translation, repression and degradation of TTFL transcripts and proteins (Figure 1), whereby during circadian day (circadian time [CT] 00-12), the heterodimerised transcription factors CLOCK and BMAL1 bind to E-box enhancer sequences to transactivate *Per* and *Cry* genes. Their encoded negative regulators PER and CRY progressively accumulate, heterodimerise and translocate into the nucleus to inhibit the CLOCK:BMAL1-dependent transactivation. During circadian night (CT12-24) therefore, E-box dependent transcription is halted and so *Per* and *Cry* mRNAs decline, followed by degradation of their encoded proteins. Finally, this degradation alleviates the transcriptional repression, such that by CT24/00 transactivation can start again, generating a cycle that repeats on a ~24 hours basis.

Additional interlocking feedback loops are formed in which transcription factors that are regulated by the TTFL also become inputs to the TTFL, creating a re-entrant motif that confers accuracy and robustness to the overall oscillation. These accessory transcription factors control gene expression by acting at a variety of response elements (REs), with the result that, as the TTFL progresses, the transcriptional drive through E-boxes and these REs undergoes high-amplitude periodic activation. This in turn allows the TTFL to drive rhythms in numerous downstream clock-controlled genes (CCGs), which sit outside the timing loop itself. In addition to this, REs present within *Per* and *Bmal1* that are sensitive to cAMP/Ca^2+^ signalling, glucocorticoid hormones or temperature allow extracellular cues to become inputs to the TTFL clock, stabilising internal synchrony (Brown, Zumbrunn, Fleury-Olela, Preitner and Schibler 2002). The overall effect, therefore, is of a pervasive, highly stereotyped programme of anticipatory gene expression and underlying chromatin remodelling that adapts cellular functions to circadian time (Koike and others 2012). Indeed, animal studies indicate that ~40% of protein-coding genes are under circadian control across tissues (Zhang, Lahens, Ballance, Hughes and Hogenesch 2014). Notably, mutations of the TTFL are associated with familial sleep disorders, the most obvious ones being mutations that alter the period of the TTFL through the disruption of PER stability (Toh and others 2001) (Meng and others 2008) (Chong, Xin, Ptacek and Fu 2018) or CRY localisation (Patke and others 2017) (Smyllie, Campbell, McManus, Patton and Hastings 2024). Moreover, there are emerging genetic links between disruption of the TTFL and impaired mental health (Walker, Walton, DeVries and Nelson 2020).

Elucidation of the TTFL transformed the study of SCN function by enabling the development of genomic and virally encoded fluorescent (Smyllie and others 2022; Smyllie and others 2016; Yang and others 2020) and bioluminescent reporters (Shan and others 2020; Yoo and others 2004) based on TTFL components. These reporters allow long-term, real-time monitoring of circadian transcription and protein expression in the *ex vivo* SCN organotypic slice, revealing a highly robust “molecular clock in a dish” with a precise temporal order: PER2 peaks at CT12, whereas CRY1 and BMAL1 protein levels peak at ~CT18. When combined with optical reporters of cellular activity, including neuronal membrane potential and the extracellular concentrations of neurochemicals, it is possible to acquire a comprehensive window onto the cell-autonomous and circuit-level rhythms in the SCN.

At the cellular level, peak electrical firing in mid-circadian day (CT06) is accompanied by depolarisation and elevated intracellular Ca^2+^ ([Ca^2+^]_i_) in neurons (Colwell 2011). This is paralleled by a circadian surge of cAMP/Ca^2+^ RE-dependent transcription, likely driven by firing-dependent changes in [Ca^2+^]_i_ (Brancaccio, Maywood, Chesham, Loudon and Hastings 2013). Through the cAMP/Ca^2+^ REs in the *Per* genes (Travnickova-Bendova, Cermakian, Reppert and Sassone-Corsi 2002), this again allows an output of the TTFL, a rhythm in electrical firing, to become an input to it, thereby enhancing its overall robustness. From the end of circadian day (CT12) electrical firing of neurons has subsided and the expression of *Per* genes declines. The reciprocal interplay between the TTFL and electrical activity of individual SCN neurons is therefore central to SCN time-keeping and, consequently, electrical silencing of SCN neurons with tetrodotoxin reduces TTFL amplitude and stability (Yamaguchi and others 2003). Beyond the individual neurons, however, the SCN has the ability to sustain circadian coherence across its own circuit and ultimately across the whole organism. This therefore begs the question: what makes it so powerful as a clock, and how can it regulate the daily activities of untold numbers of cells, day after day?

### Time-keeping by the SCN neuronal network: a story of neuropeptides

**Figure 2:**
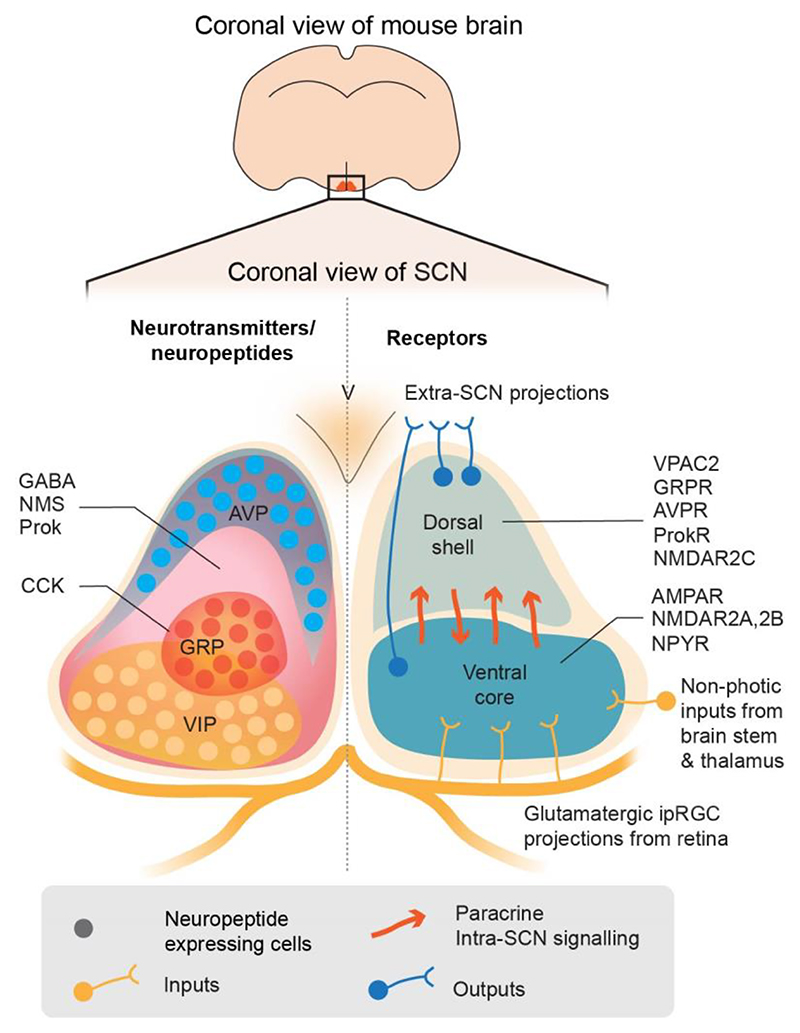
Neuroanatomical organisation of the SCN. (Upper) Position of the SCN within the coronal view of a mouse brain. (Lower) Schematic coronal view of SCN organisation showing the anatomical distribution of neurotransmitters and neuropeptides (left), and receptors (right) across the SCN. The paired SCN sit above the optic chiasm, flanking the third ventricle (V). The SCN can be coarsely divided into two anatomical subdivisions, the ventral core (right, dark blue) and the dorsal shell (right, light blue). Neuropeptides and neurotransmitters of the SCN (left): GABA-, neuromedin-S (NMS)- and prokineticin (Prok)-expressing neurons are distributed across the whole of the SCN (pink shading), overlapping with other cell populations. Vasoactive intestinal polypeptide (VIP)-(orange shading), gastrin-releasing peptide (GRP)- and cholecystokinin (CCK)-(red shading) expressing neurons are restricted to the ventral core, while arginine vasopressin (AVP) neurons (blue) are restricted to the dorsal shell of the SCN. Neuropeptide and neurotransmitter receptors of the SCN (right): neurons expressing ionotropic glutamatergic AMPA and NMDA receptors are localised to the core of the SCN where they receive entraining glutamatergic signals that are released from intrinsically photoreceptive retinal ganglion cell (ipRGC) terminals arriving via the retinohypothalamic tract (RHT) and the optic chiasm (light orange delineation and terminals). This region also expresses neuropeptide Y receptors (NPYR) that receive non-photic inputs arriving from the thalamus (light orange terminal). VPAC2, GRPR, AVPR and ProkR neuropeptide receptors as well as NMDA receptors containing the NR2C-subunit are expressed in the shell of the SCN, coupling the core and shell primarily by neuropeptidergic signals (dark orange arrows). SCN outputs are conveyed from the shell and core of the SCN, predominantly by GABAergic and neuropeptidergic signals (blue terminals).

Although all SCN neurons express the inhibitory neurotransmitter GABA, the SCN is not a homogeneous network (Figure 2). It consists of a retinorecipient core region that contains neurons expressing the neuropeptides vasoactive intestinal polypeptide (VIP) and gastrin-releasing peptide (GRP) alongside AMPA- and NMDA-type (NR2A, NR2B) ionotropic glutamate receptors. The light-induced activation of these receptors by glutamatergic RHT terminals depolarises the VIP and GRP neurons, increasing their firing rate and [Ca^2+^]_i_. This leads to acute induction of *Per* genes via their Ca^2+^ REs, such that their cell-autonomous TTFL is delayed at dusk or advanced by dawn light. The surrounding SCN shell region contains neurons that express arginine vasopressin (AVP) and other neuropeptides, alongside a particular sub-type of NMDA receptor (NR2C), as well as receptors for VIP (VPAC2) and GRP (GRPR). Through these receptors, neuropeptides released by core neurons can synchronise the TTFL of shell neurons, again via signalling pathways that converge on Ca^2+^ REs in the *Per* genes (Hamnett, Crosby, Chesham and Hastings 2019). These serial actions maintain temporal coherence of electrical activity and metabolism across the SCN and its temporal alignment to the light-dark cycle.

In addition to entrainment, neuropeptide signalling has a critical role in the steady state oscillation of the SCN in the absence of entraining cues. Although the individual cell-autonomous TTFL clocks are synchronised across the circuit, they are not simultaneously active: the intensity and relative phase of peak *Per* and *Cry* expression vary systematically across the SCN. This creates a stereotypical wave of gene expression that moves from the dorsomedial to the ventral and then to the dorsolateral regions of the SCN. This wave can be interpreted as the passing of a temporal signal, moving in relay between sequential neuronal sub-populations. For example, the circadian rhythms of cellular activity of VIP cells in the SCN core, are advanced by ~2 hours relative to those of their target cells expressing the VPAC2 receptor in the SCN shell (Patton and others 2020). This suggests that VIP-to-VPAC2 signalling constitutes one stage of the serial flow of temporal information through the SCN network. Consistent with this, optogenetic activation of VIP neurons can induce *Per* expression in the shell and shift the phase of the overall SCN oscillation (Mazuski and others 2018; Patton and others 2020), whilst genetic loss of VIP or VPAC2 desynchronises and destabilises the cell-autonomous clocks across the entire SCN (Aton, Colwell, Harmar, Waschek and Herzog 2005; Maywood and others 2006). This disorganisation can be rescued *ex vivo* when VIP-deficient SCN slices are co-cultured with VIP-proficient SCN: an effect mediated by paracrine signalling by VIP (Maywood, Chesham, O’Brien and Hastings 2011). Furthermore, in the absence of AVP-dependent signalling in mice deficient in AVP receptors, SCN circadian oscillations of electrical firing and TTFL activity persist, as does circadian behaviour, but they are more easily disrupted by treatment with TTX and by altered lighting cycles, respectively (Yamaguchi and others 2013). Thus, network level robustness requires VIP- and AVP-mediated signalling, which binds together the cell-autonomous TTFLs of the neuronal sub-populations within the SCN network by controlling electrical firing and *Per* gene expression.

The advent of single-cell RNA sequencing has provided a more comprehensive view of the cellular complexity and network topology of the SCN (Morris and others 2021; Wen and others 2020), revealing additional cell clusters enriched in either neuropeptides or neuropeptide receptor transcripts, including cholecystokinin (CCK), somatostatin (SST), Prokineticin (Prok) and neuromedin-U/S (NMU/S). At a transcriptional level, these and the corresponding VIP-an AVP-signalling axes are expressed in circadian day and decline during circadian night (Morris and others 2021), implying a TTFL-dependent programme of daytime assembly and night-time dismantling of intra-SCN peptidergic signalling networks. This is consistent with the daytime release of VIP and other neuropeptides, but also raises the question of what timing signals hold the circuit together and sustain neuronal inactivity in circadian night: what complements daytime activation by neuropeptidergic cues? GABA is an inhibitory transmitter in the SCN (Patton and others 2023), but SCN neurons fire at very low rates in circadian night and so spontaneous synaptic GABAergic signalling will be limited at that time. Furthermore, intersectional studies that have disrupted GABAergic signalling in SCN neurons indicate that synaptically released GABA may be more important as a timing signal for downstream neural targets outside the SCN, rather than as a mediator of intra-SCN time-keeping (Ono, Honma, Yanagawa, Yamanaka and Honma 2019). A growing body of evidence now points to a significant role for nocturnally active astrocytes, and their cell-autonomous TTFL clock, as an integral component of the SCN time-piece.

### Astrocytes, neuronal signalling and clocks

Traditionally, astrocytic regulation of neuronal signalling has centred around the tripartite synapse, in which pre- and post-synaptic neuronal elements are ensheathed by surrounding astrocytic projections, conferring structural organisation to the synapse as well as allowing astrocytes to respond to neuronal signals. Conceptually, this would enable astrocytes to regulate the local neurochemical environment by the uptake of neurotransmitters and the release of gliotransmitters (Araque and others 2014; Verkhratsky, Parpura, Li and Scuderi 2021) and thereby regulate neuronal excitability (Verhoog, Holtman, Aronica and van Vliet 2020). In the SCN, astrocytes undergo progressive daily changes in morphology and in the intensity of staining for the astrocytic marker glial fibrillary acidic protein (GFAP) (Lavialle and Serviere 1993). This indicates that they may be actively involved in circadian regulation by periodically unsheathing and exposing SCN neurons to synaptic inputs, with glutamatergic signalling from the RHT driving the cycle (Lavialle and others 2011). In this setting, however, information transfer remains the domain of the neurons (retinal and SCN) and not astrocytes, the neurons operating with a very rapid time course and their signalling being spatially restricted. Beyond the tripartite synapse hypothesis, however, astrocytes also modulate the flow of information across broader neuronal networks and, given their lack of electrical excitability and morphology, they are well placed to act slowly and progressively over much larger temporal and spatial domains (Haydon 2017). Indeed, it has been proposed that astrocytes establish a landscape in which information-processing by neuronal networks is influenced by, and made more appropriate to, external and internal contexts (Murphy-Royal, Ching and Papouin 2023). This extended suite of cellular/ network processes controlled by astrocytes therefore indicates that astrocytes may be more active modulators of neuronal circuits than the tri-partite synapse suggests.

With the development of genetic access to astrocytes, it has become possible to move away from observational studies and to explore their causal roles across the brain in the modulation of complex, network-driven behaviours (Nagai and others 2021). This has been augmented by transcriptomic approaches that have revealed the diversity of astrocytes across the brain (Ben Haim and Rowitch 2017). Importantly, because a single astrocyte can connect to multiple synapses, and since astrocytes can operate as a syncytial network via gap junction contacts, they can, individually or as a network, integrate neuronal information from an extensive area. Equally, they can regulate the activity states of many neurons across their local territory (Araque and others 2014). These properties make astrocytes important contributors to the control of sleep and other arousal states. Their activity changes dynamically across the cycle of sleep and wakefulness and they may encode homeostatic sleep drive via their intracellular signalling pathways. They may also release sleep-inducing molecules to regulate brain activity and sleep architecture (Ingiosi and Frank 2023). For example, compromise of astrocytic vesicular signalling can impair recovery sleep following sleep deprivation, possibly by limiting adenosine release during imposed wakefulness and thereby reducing the drive for subsequent recovery sleep (Haydon 2017). More acutely, temporally controlled optogenetic activation of astrocytes can trigger slow oscillations in the cerebral cortex (Poskanzer and Yuste 2016), possibly by modulating glutamatergic signalling. Given the slow-acting temporal regulation of neuronal activity by astrocytes across the brain, do they act in the circadian domain?

**Figure 3:**
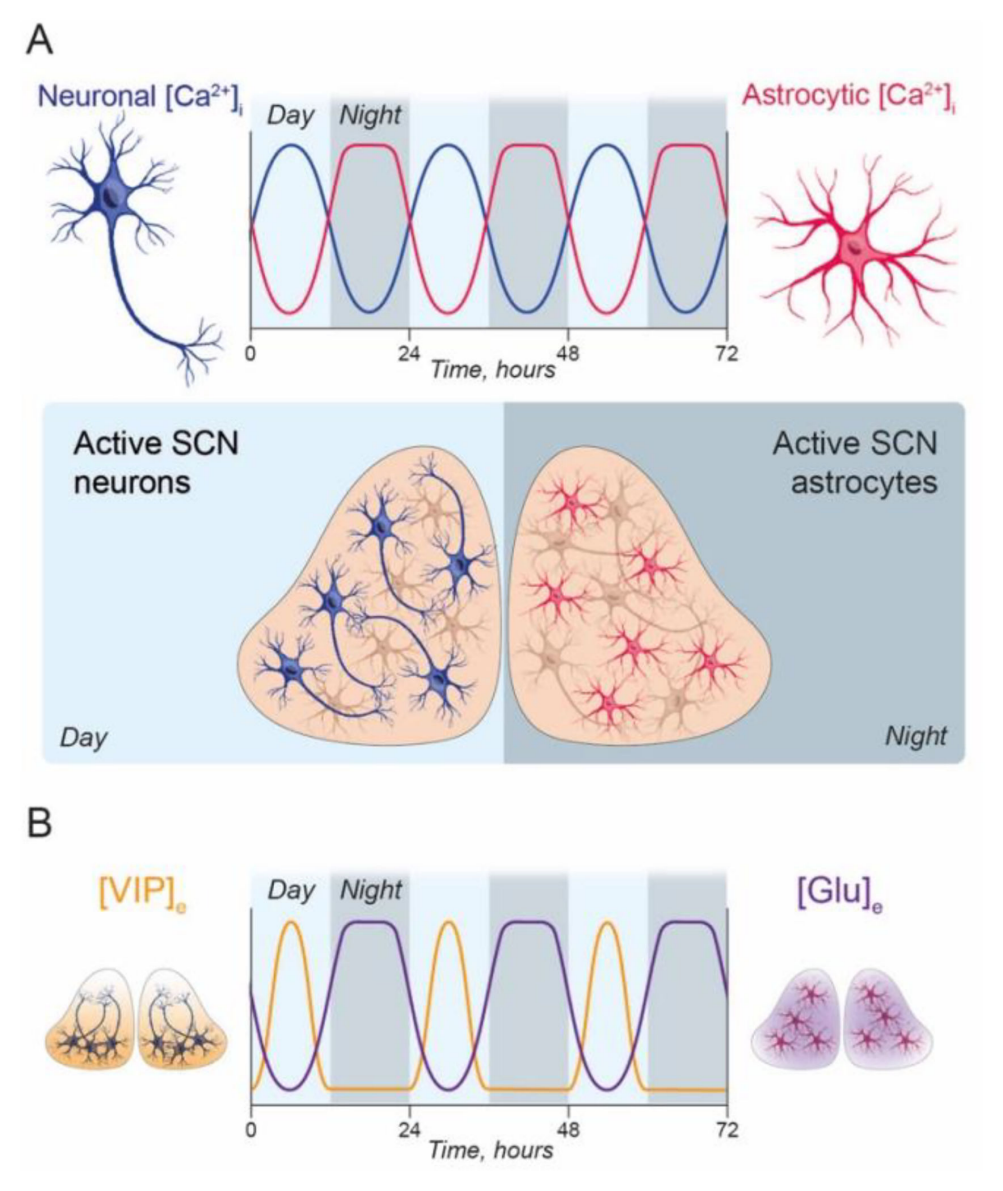
Differential circadian phasing of neurons and astrocytes in the SCN. A. (Upper) Schematic representation of the relative phasing and waveforms of oscillations in intracellular calcium levels ([Ca^2+^]_i_) of SCN neurons (blue) and SCN astrocytes (pink). Peaks in [Ca^2+^]_i_ fall during mid-circadian day (light grey bars, *Day*) for neurons and mid-circadian night (dark grey bars, *Night*) for astrocytes. (Lower) Based on the relative phasing of [Ca^2+^]_i_ in these cell-types, neurons are positioned as the day-time active component of the SCN network while astrocytes are the night-time active component. B. Schematic representation of the relative phasing and waveforms of oscillations in signalling molecules in the extracellular space. VIP ([VIP]_e_, orange) is released by neurons whilst glutamate ([Glu]_e_, purple) is from astrocytes. Similar to the relative activities of the cells (A, upper), the timing of signals in the extracellular space are split between circadian day (light grey bars, *Day*) and night (dark grey bars, *Night*) and are co-phasic with the activity of their source cell-type, emphasising the daily alternation of cellular functions and signalling pathways.

Considering that most cells in the body contain a functional circadian clock, it is unsurprising that astrocytes can also express cell-autonomous TTFL rhythms. These were first identified in cultured cortical astrocytes (Prolo, Takahashi and Herzog 2005), and confirmed in *ex vivo* SCN slices expressing astrocyte-specific TTFL reporters (Brancaccio and others 2019; Tso and others 2017). Conditional expression of a *Cry1-Luciferase* transcriptional reporter suggested that the TTFL of SCN astrocytes was phase-lagged by a few hours relative to SCN neurons, and this phase-difference was seen more clearly when [Ca^2+^]_i_ was recorded simultaneously in astrocytes and neurons. As noted above, [Ca^2+^]_i_ peaks in mid-circadian day in SCN neurons but in SCN astrocytes it peaks in the middle of circadian night (CT18), in antiphase to neuronal [Ca^2+^]_i_ (Figure 3A). Furthermore, it has a distinctive waveform with a broad nocturnal peak and sharply defined nadir, such that it is the inverse of the waveform of neuronal [Ca^2+^]_i_. The nature and functions of [Ca^2+^]_i_ signalling in astrocytes are complex and incompletely understood (Bazargani and Attwell 2016; Khakh and McCarthy 2015), and as astrocytes are non-excitable, these changes in [Ca^2+^]_i_ are not driven by electrical activity. The source, therefore, is more likely linked to release of Ca^2+^ from intracellular stores, or through activation of G-protein coupled receptors. They therefore represent the metabolic, rather than electrical, state of the astrocytes (Semyanov, Henneberger and Agarwal 2020). Nevertheless, this difference in phasing between SCN astrocytes and neurons suggests mutually antagonistic coupling, whereby daytime signals released by electrically active neurons suppress astrocytic function, whereas silencing of neurons and their neuropeptidergic networks during circadian night allows astrocytes to become active, possibly inhibiting neuronal activity. Indeed, factors released by astrocytes (e.g., glutamate) and neurons (e.g., VIP) exhibit similar antiphasic patterns in the SCN extracellular space, consistent with their alternating cellular activity (Brancaccio, Patton, Chesham, Maywood and Hastings 2017; Patton and others 2023) (Figure 3B).

So, what is the evidence that astrocytes contribute to SCN time-keeping? Direct metabolic challenge of astrocytes with fluorocitrate, which compromises mitochondrial aconitase activity but is not taken up by neurons, reversibly disrupts and attenuates their circadian peak of [Ca^2+^]_i_ in organotypic SCN slices (Patton, Smyllie, Chesham and Hastings 2022). Accompanying this, the circadian TTFL rhythms, as reported by PER2-driven bioluminescence, are damped, suggesting that astrocytes participate in network-level circadian timekeeping, potentially by boosting or at least sustaining the circadian TTFL programme of neurons.

### The cell-autonomous TTFL clock of SCN astrocytes and the control of circadian rhythms

Astrocytic function is therefore necessary for SCN time-keeping, but a more demanding question is: do astrocytes truly contribute to the creation of circadian information? To answer that, it is necessary to manipulate the cell-autonomous TTFL of astrocytes and examine the effects on circadian organisation. Global astrocytic deletion of *Bmal1* to render them “clockless” did not make mice behaviourally arrhythmic but it did lengthen the circadian period of rest/activity behaviour (Barca-Mayo and others 2017; Tso and others 2017). A comparable period lengthening was observed in the SCN slice *ex vivo*, suggesting that when the neuronal clock is functional, the astrocyte clock is dispensable for rhythmicity. In the absence of circadian outputs from the astrocytes, however, the neuronal clock runs more slowly, which may suggest that, ordinarily, it receives stimulatory signals from astrocytes.

**Figure 4:**
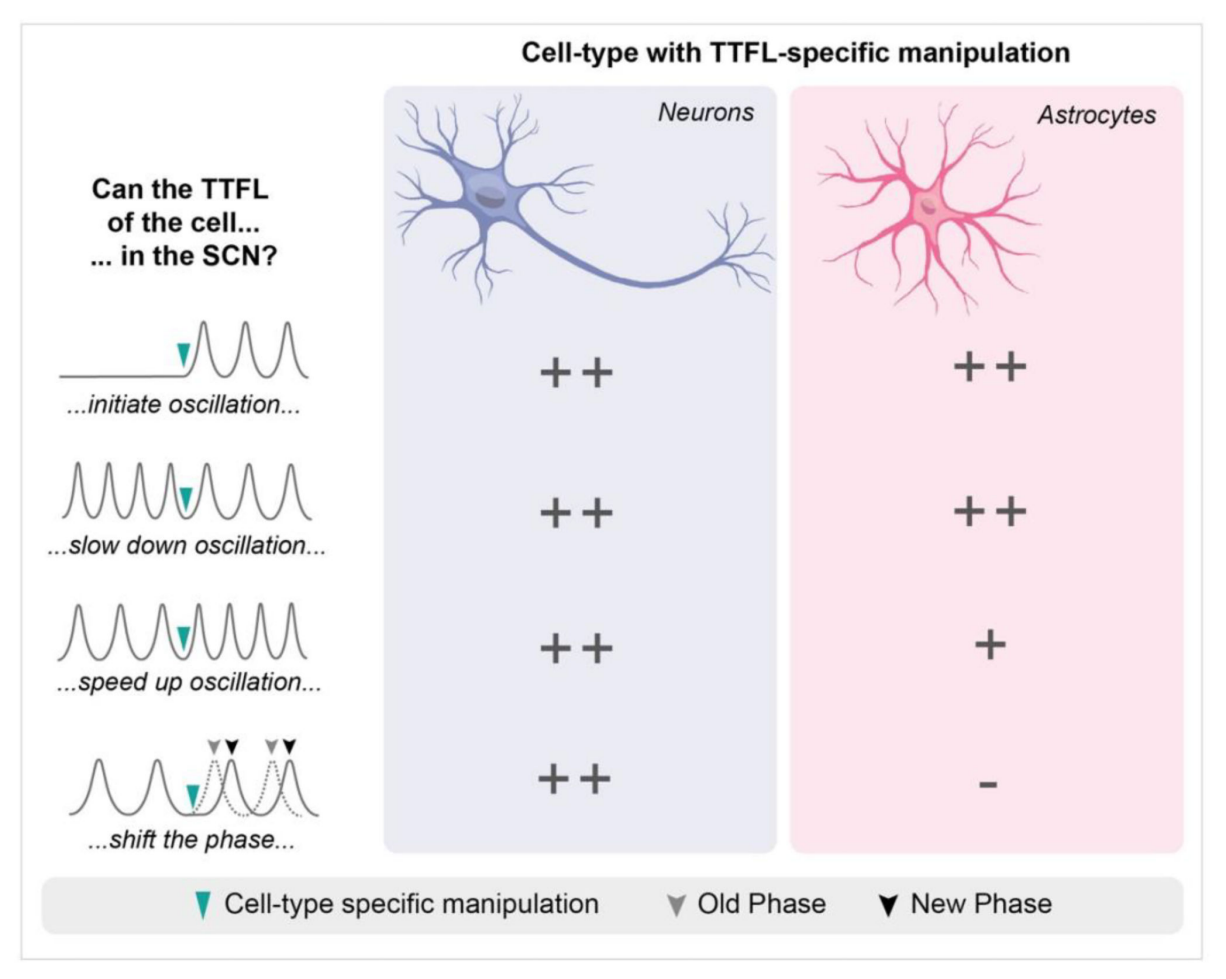
Comparison of the contributions of the cell-autonomous clocks of neurons and astrocytes to SCN timekeeping. Summary of the contributions of neurons and astrocytes to SCN timekeeping, tested using cell-type specific manipulation of TTFL function in *ex vivo* SCN slices and *in vivo*. The results of manipulation are indicated as: ++ full control of this aspect of timekeeping, + some control of this aspect of timekeeping, **-** no control of this aspect of timekeeping across the SCN network.

An alternative experimental approach is to activate the cell-autonomous clock of the two cell types separately, and this can be achieved by using viral vectors to express CRY protein in the SCN of arrhythmic CRY-deficient mice. When CRY is targeted solely to SCN neurons, appropriate SCN TTFL and behavioural rhythms are initiated (Maywood and others 2018) (Figure 4). The cell-autonomous clock of astrocytes is therefore not necessary for SCN circadian function, whereas the cell-autonomous clock of neurons is sufficient for SCN circadian function. This does not, however, disprove a role for the astrocyte clock under normal circumstances. Indeed, when global CRY deficiency is genetically complemented solely in SCN astrocytes, such that these astrocytes contain the only functional clock in the mouse, they can nevertheless initiate circadian rhythms of [Ca^2+^]_i_ in SCN neurons and TTFL rhythms across the neuronal network. Even more startling, they can initiate circadian behavioural rhythms *in vivo* (Brancaccio and others 2019; Patton and others 2023; Patton, Smyllie, Chesham and Hastings 2022). These results invert the necessity vs. sufficiency argument applied to neuronal clocks, and demonstrate that astrocytic clocks are sufficient for SCN time-keeping. Therefore, astrocytes are active partners in the SCN network.

This partnership in circuit-level time-keeping can also be tested in temporally chimeric SCN, in which neuronal and astrocytic TTFLs have different intrinsic periods created by intersectional genetic approaches. For example, the CK1-epsilon *Tau* mutant allele (CK1*ε*^Tau^) destabilises PER2 and accelerates the TTFL to a period of ~20 hours in homozygotes. When it is deleted, however, the TTFL period reverts back to wild-type, i.e., ~24 hours (Meng and others 2008). Conditional deletion of this mutant allele from SCN neurons or astrocytes, will therefore, reveal the capacity of these two cell-types to control the period of SCN-encoded time. Unsurprisingly, its deletion in SCN neurons alone lengthened ensemble TTFL period and circadian behaviour. What is remarkable, however, is that its deletion from SCN astrocytes had the same effect (Brancaccio, Patton, Chesham, Maywood and Hastings 2017; Tso and others 2017). Moreover, astrocytes achieved this dominant control of SCN period without disrupting overall circuit or behavioural coherence (Figure 4), i.e., the astrocyte-dominated clock keep functioning normally.

For both SCN neurons and astrocytes to be able to slow one another in this way, the reciprocal signalling between them must be substantial. Presumably, it is mediated by an inhibitory drive because it is easier to slow down the clock of a different cell by inhibition rather than by excitation (Patton, Smyllie, Chesham and Hastings 2022). When the TTFL period of neurons is shortened through conditional expression of CRY2, the speed of the SCN network accelerates accordingly. In contrast, the same manipulation of astrocytes does not affect SCN period: neurons remain dominant (Patton, Smyllie, Chesham and Hastings 2022) (Figure 4). This may reflect the differences in respective cell numbers in the SCN (10,000 neurons vs. 3,000 astrocytes), but it may also reflect more powerful signalling from neurons to astrocytes.

The final aspect of circadian timekeeping is the phase-control that mediates adaptive entrainment to external and internal cues. Neurons are very adept at this, receiving a number of synaptic inputs, most notably from the RHT, which allow them to align their cellular activity and TTFL to solar time. Correspondingly, direct chemogenetic or optogenetic activation of SCN neurons (Jones, Simon, Lones and Herzog 2018; Patton, Smyllie, Chesham and Hastings 2022) can shift the phase of TTFL gene expression rhythms *ex vivo* and circadian behaviour *in vivo*. In contrast, even though direct chemogenetic manipulation of astrocytes via G_i_- or G_q_-coupled receptors stimulates calcium signalling pathways, it is unable to alter the phase of the ongoing SCN oscillation (Patton, Smyllie, Chesham and Hastings 2022). This contrast between neurons and astrocytes may reflect the fact that entrainment relies on responsiveness to synaptic inputs from the retina and brain stem that are indicative of solar time and arousal state, respectively. This information is not conveyed to astrocytes and so it is unsurprising that they lack any capacity to shift network phase contingent on these signals. Nevertheless, even though astrocytes may not be able to alter phase acutely, they may modulate and augment the network responses to phase-shifting stimuli. For example, reciprocal co-operation between astrocytes and neurons can induce phase advances through neuron-to-astrocyte activation of cannabinoid receptors, which then feeds back to activate neuronal adenosine receptors (Hablitz, Gunesch, Cravetchi, Moldavan and Allen 2020). In conclusion, therefore, SCN astrocytes can be elevated from passive participants in SCN network timekeeping to active participants. In temporally chimeric SCN they can slow the period of the oscillation in direct competition with neurons, and they can initiate and organise neuronal rhythmicity, both *ex* and *in vivo*, when they are the only cells containing a functional TTFL. This raises the question of how the respective TTFLs of SCN neurons and astrocytes signal time to each other.

### Neurochemical basis of astrocytic control of SCN network time-keeping

Astrocytes actively release neurotransmitters and neuropeptides alongside neuromodulators, hormones and metabolites (Araque and others 2014; Verkhratsky, Parpura, Li and Scuderi 2021), although the mechanisms of release are highly contested, with a viewpoint that Ca^2+^-dependent glutamate exocytosis from astrocytes does not occur under physiological conditions (Fiacco and McCarthy 2018). This begs the question of what mechanism may actually regulate the release of such signals, although more recent technical developments suggest that Ca^2+^-dependent exocytosis of factors such as glutamate may indeed contribute, at least in specialised astrocytes (de Ceglia and others 2023) and sub-regions of the cell (Bazargani and Attwell 2016). The candidate gliotransmitters in the SCN include ATP (Burkeen, Womac, Earnest and Zoran 2011; Marpegan and others 2011) adenosine (Hablitz, Gunesch, Cravetchi, Moldavan and Allen 2020) and glutamate, which is of interest in the SCN for a number of reasons. First, glutamate levels in the extracellular space ([Glu]_e_) oscillate on a circadian basis in SCN *ex vivo* slices (Brancaccio, Patton, Chesham, Maywood and Hastings 2017). Furthermore, blockade of astrocytic glutamate transporters sustains elevated [Glu]_e_ and damps the TTFL of the SCN slice, suggesting that extra-synaptic glutamatergic cues are important for neuronal rhythmicity. Importantly, the profile and phase of [Glu]_e_ are precisely in register with astrocytic [Ca^2+^]_i_ suggesting that it is controlled by astrocytes (Brancaccio, Patton, Chesham, Maywood and Hastings 2017). This is supported by several observations: first, the neurons of the SCN are GABAergic and are therefore incapable of releasing glutamate; second, the oscillation of [Glu]_e_ is antiphasic to neuronal activity (Figure 3B), and so neuronal activity cannot drive it. In addition, [Glu]_e_ oscillations are acutely disrupted by pharmacological manipulation of astrocyte-specific release pathways (e.g., connexin-43 hemichannels) (Brancaccio and others 2019). Finally, initiation of the SCN network oscillation by activation of the astrocytic clock is compromised when [Glu]_e_ release is disrupted. The circadian regulation of [Glu]_e_ therefore provides a key mechanism for astrocytes to transfer the temporal information that is generated by their own cell-autonomous clock onwards to SCN neurons.

**Figure 5:**
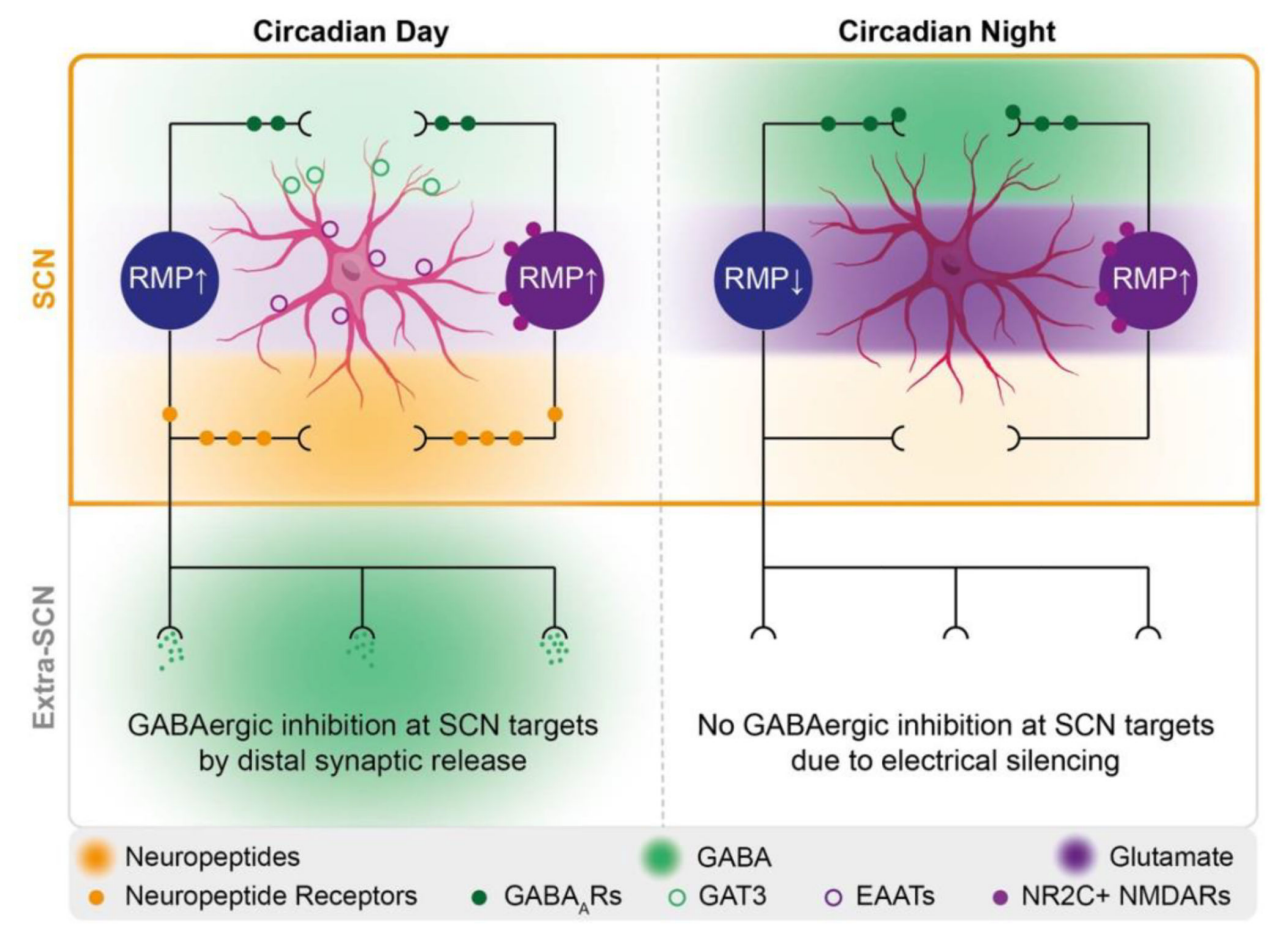
Network model of SCN neuronal-astrocytic communication. Schematic network model of how SCN neuronal-astrocytic communication sustains and controls network-level timekeeping. During circadian day (Left) when neurons are electrically active (blue/purple), a depolarised resting membrane potential (RMP↑) is maintained by uptake of locally released synaptic GABA (green shading) within the SCN via GABA transporter 3 (GAT3, green circles) located on SCN astrocytes (pink). Otherwise, the GABA would suppress neuronal activity. Daytime neuronal activity facilitates paracrine release of neuropeptides local to the SCN (orange shading), which further promotes SCN neuronal electrical activity and intracellular signalling cascades via neuronal neuropeptide receptors (filled orange circles). During this time, low extracellular glutamate levels (purple shading) are maintained by uptake through astrocytic excitatory amino-acid transporters (EAATs, light purple circles). Daytime electrical activity allows SCN neurons to release GABA synaptically at distal sites, facilitating daily control of behaviour and physiology. During circadian night (Right), astrocytes no longer express GAT3 and this allows GABA (green shading) to accumulate outside SCN neurons where it can activate GABA_A_ receptors (filled green circles) leading to neuronal hyperpolarisation (RMP↓) and electrical silencing. Alongside this, SCN astrocytes release glutamate (purple shading) and consequently, in the nocturnal absence of EAAT activity, high glutamate levels are maintained. This extracellular glutamate is sensed by a subset of SCN neurons (purple) that express NR2C-containing NMDA receptors (filled purple circles) allowing them to become active during the night to augment GABA release and accumulation. This, coupled with the night-time reduction in neuropeptide and neuropeptide-receptor expression, results in the bulk of SCN neurons being electrically silenced at night. This night-time electrical quiescence has two effects: first, network input to the TTFL is curtailed allowing the repressive phase of the clock to progress correctly, and second, GABAergic inhibition at distal SCN sites is relieved at night, and this circadian change in distal GABAergic release allows the SCN to temporally coordinate the activities of its downstream targets.

How is [Glu]_e_ sensed by SCN neurons? As noted earlier, the retinorecipient core neurons of the SCN express ionotropic NMDARs that contain NR2A and NR2B subunits (Colwell 2011). These receptors only respond to glutamate when its presence is coincident with depolarisation of the neuron, which itself is caused by co-expressed ionotropic AMPARs. This mechanism is well characterised in the context of photic entrainment but cannot explain how night-time hyperpolarised neurons sense astrocyte-derived glutamate. Yet, the SCN also expresses NMDARs that contain the NR2C subunit, which is enriched in the SCN shell (Brancaccio, Patton, Chesham, Maywood and Hastings 2017). The presence of this subunit allows glutamatergic activation of the receptor without depolarisation (Paoletti, Bellone and Zhou 2013), making it ideally suited to sensing glutamate during the night, when SCN neurons are hyperpolarised. This supports a model whereby extracellular glutamate released from the astrocytes is sensed by the sub-population of neurons expressing NR2C, and this sustains elevated presynaptic calcium levels. Consequently, this enhances the tonic release of GABA from these neurons, in a mechanism that is independent of action potential firing, and this in turn suppresses the firing rate of neurons across the network. Consistent with this model, nocturnal blockade of NR2C-containing receptors results in depolarisation of SCN neurons across the network, and this is associated with a decrease in pre-synaptic [Ca^2+^]_i_ reflecting the reduced activation of pre-synaptic NR2C receptors (Brancaccio, Patton, Chesham, Maywood and Hastings 2017) (Figure 5).

The model predicts, therefore, increased GABAergic tone during circadian night and consistent with this, GABA levels in the SCN extracellular space ([GABA]_e_) are highly rhythmic and co-phasic with [Glu]_e_, both peaking at night (Patton and others 2023). They are therefore antiphasic to neuronal activity: an unanticipated, paradoxical relationship if the activity of the GABAergic SCN neurons were the sole determinant of [GABA]_e_. Resolving this paradox, it is now clear that the [GABA]_e_ rhythm is controlled by the astrocytic clock. In a genetically clockless SCN, the [GABA]_e_ rhythm is lost, but can be restored by activating the TTFL specifically in astrocytes (Patton and others 2023). The rhythm is driven by the activity of the astrocyte-specific GABA transporter (GAT) GAT3. Indeed, pharmacological blockade of GAT3 causes progressive daytime elevation of [GABA]_e_ that eventually obscures the rhythm. The elevation is accompanied by damping of the SCN TTFL in cells and across the circuit. Whether these GABA dynamics arise purely from astrocytic control of neuronal activity via uptake though GATs, or whether astrocytes are also a source for GABA release in the SCN, remains to be seen.

The model of SCN astrocyte-to-neuron signalling can now be extended (Figure 5). During circadian day, the cell-autonomous clock of astrocytes increases GAT3 expression and its activity lowers [GABA]_e_. Because GABA inhibits SCN neuronal firing via GABA_A_ receptors, this daytime reduction of [GABA]_e_ is a de-repression and allows SCN neurons to fire at peak rates, as co-ordinated by their TTFL. This in turn supports daytime paracrine neuropeptidergic signalling, synchronising TTFL activity across the circuit. In circadian night, astrocytic GAT3 expression declines and [GABA]_e_ increases, suppressing neuronal activity and the associated neuropeptide release as the neuronal TTFL simultaneously dismantles the neuropeptidergic signalling axes. Thus, SCN astrocytes exert control of neuronal excitability by two, complementary pathways that converge on [GABA]_e_. First, by nocturnal enhancement of GABAergic tone mediated by astrocytically derived glutamate acting at neuronal NR2C receptors and, second, via circadian GAT3-dependent changes in GABA uptake. This clears the way for SCN neurons to fire during circadian day, as they are driven by their cell-autonomous TTFL, and to enter electrical quiescence during the night when neuropeptidergic signalling is dis-assembled. In this way, the circadian information generated by the cell-autonomous TTFL of astrocytes directs the SCN neuronal network. That is to say, the source of information in the circuit is entirely astrocytic.

## Conclusions

The model presented above is qualitatively different from the conventional view that astrocytes simply modulate the processing of information generated by neurons, but have no intrinsic information-generating capability. It is clear that astrocytes are active participants in SCN timekeeping because circadian information generated solely by their cell-autonomous TTFL can drive rhythms in SCN neurons and in circadian behaviour. Second, when astrocytes and neurons both have functional clocks but with different periods, astrocytes can impose their cell-intrinsic, TTFL-defined period on neurons. In both situations astrocytes are more than simple modulators of neurons. Rather, the system is made to behave differently from how it would operate were it to be based on neuronally generated information alone. An important caveat is that some of these conclusions are drawn from work on astrocytes in organotypic SCN explants, which may not reflect the function state of the astrocytes in an intact animal (Semyanov, Henneberger and Agarwal 2020). It is worth noting, however, that there are tight correlations between the findings from this work *ex vivo* and the same manipulations *in vivo* regarding astrocytic control over both circadian period and the initiation of rhythms (Brancaccio, Patton, Chesham, Maywood and Hastings 2017) (Brancaccio and others 2019). This suggests that astrocytic circadian function persists in the SCN network equally between *ex vivo* and *in vivo*. More generally, it raises the question of whether other brain activities operating over prolonged time-bases suitable for astrocytic computation, such as sleep and transitions between arousal state, are also directed by information-processing in astrocytes. A starting point to examine this would be to explore the role of astrocytes in local brain clocks, for example in the cerebral cortex and cerebellum (Abe and others 2002). Does selective, rather than global, manipulation of the astrocytic TTFL affect local circuit functions?

A second question is the generality of the neurochemical mechanisms proposed. To transfer their circadian information, the TTFL of SCN astrocytes controls circadian changes in [GABA]_e_ that facilitate daytime neuronal activity and nocturnal neuronal quiescence. This rhythm of neuronal activity is the critical output of the SCN because synaptic release of GABA at extra-SCN targets conveys time cues to local brain clocks and neuroendocrine pathways. The devolution of circadian control of [GABA]_e_ to astrocytes is an attractive design feature because it dissociates [GABA]_e_ from the firing rate of GABAergic neurons. Specifically, the daytime reduction of [GABA]_e_ allows SCN neurons to fire even though they are embedded in a GABAergic circuit in which local synaptic release of GABA would otherwise be expected to suppress the network. In parallel, daytime network activity is also promoted by neuropeptidergic signalling, directed by the neuronal TTFL. Together, this allows daytime synaptic GABA release and nocturnal quiescence at distal sites to control circadian behaviours and physiology. It remains to be seen whether this devolved role of astrocytes in regulating [GABA]_e_ is a general property of other GABAergic circuits in which synaptic firing has to be sustained for several hours, as is the case in the SCN. For example, GAT3 expression in the cerebral cortex is dependent on BMAL1 (Barca-Mayo and others 2017) and astrocytic deletion of *Bmal1* causes levels of GABA in the CSF to rise because of the absence of GAT3. Moreover, co-culture experiments indicate that, as in the SCN, GABA mediates astrocytic synchronisation of the TTFL of cortical neurons (Giantomasi and others 2023). Perhaps the nature of circadian time-keeping is well suited to regulation by astrocytes, insofar as it is dependent on progressive, long-acting paracrine signals operating at distance. More broadly, the paracrine signalling of circadian time by neuropeptides, glutamate, GABA and purinergic signals may be viewed as volume transmission, which is also a particular feature of monoaminergic innervation across the forebrain and in which local astrocytic control is an important regulator (Gianni and Pasqualetti 2023). Are there mechanistic parallels here with the SCN, not least in the circadian modulation of arousal, mood and cognition?

Our current model of astrocytic control of the SCN network posits a co-ordinated interplay between the uptake and release of GABA and astrocyte-derived glutamate. Although there is controversy surrounding the ability of astrocytes to release glutamate actively, this discussion is generally focused on fast-acting timescales, comparable to glutamatergic neurotransmission (Fiacco and McCarthy 2018) (Bazargani and Attwell 2016). This may not be the relevant comparator and, even though specialised astrocytes have been identified recently within the hippocampus that release glutamate via vesicular mechanisms (de Ceglia and others 2023), the model of fast-acting glutamatergic gliotransmission is not required by our SCN model. Rather, as with GABA, we expect glutamate to act on a more prolonged circadian time-scale as indicated by the [Glu]_e_ dynamics (Figure 3B) and its release is not necessarily via vesicular mechanisms, but possibly by hemi-channels (Brancaccio and others 2019).

This leaves the observations on SCN astrocytes in a potentially interesting position that may inform the debate around the role of astrocytes in controlling extracellular glutamate. A further aspect is the role of the NR2C NMDAR-subunit that responds to this glutamate. This sub-unit is highly expressed in neurons of the cerebellum, thalamus, olfactory bulb and basal ganglia (Hansen, Yi, Perszyk, Menniti and Traynelis 2017) (Ravikrishnan and others 2018), raising the possibility that it contributes as a sensor of glutamatergic paracrine cues in brain areas beyond the SCN.

Alongside these astrocyte-to-neuron signalling axes, we expect that other signalling axes exist within/ between astrocytes and other glial cells in the SCN that are still to be uncovered. In addition, the reciprocal neuron-to-astrocyte (and other glial cells) signals, which would act to iteratively boost network cohesion, are unknown. How does the clock of neurons convey its time-cues to the TTFL of astrocytes? Neuropeptides, especially VIP (Marpegan, Krall and Herzog 2009), are likely important but how, not least because they are released when astrocytes are inactive? Do they inhibit astrocytic activity to engage their TTFL? Determining these signalling pathways, and indeed how they integrate these cell-types into the network of the SCN, will be important for pinpointing what happens when these cellular clocks are compromised. This will be critical in enhancing our understanding of the progression of circadian dysfunction underlying pathological states, including neurodegenerative disorders (Hastings and Goedert 2013), where disturbed sleep phenotypes can be prominent, and circadian disruption of glial cells is thought to underly disease progression (McKee, Lananna and Musiek 2020).

## Box 1 Over-view of the mammalian circadian timing system: the SCN co-ordinates a distributed network of clocks.

The SCN, depicted here as the red clock symbol in a schematic sagittal view of a mouse brain, is located in the anterior hypothalamus (green). It is the principal pacemaker and co-ordinator of the mammalian circadian network, with cell types, organisation and neurochemical identity conserved across rodents, humans and other species. The foundational discoveries that identified the SCN as the principal clock driving circadian behaviour and sleep in mammals are reviewed in (Weaver 1998). Together, these studies demonstrate that the SCN clock is necessary and sufficient to support circadian behaviour, *in vivo*. To achieve this, the SCN autonomously generates circadian rhythms in neural firing, gene-expression and metabolism that persist *ex vivo* in tissue explants and dispersed cultures. It is synchronised to environmental time by direct input from intrinsically photoreceptive retinal ganglion cells (ipRGC), via the retinohypothalamic tract (RHT), its so-called photic inputs (LeGates, Fernandez and Hattar 2014). It also receives non-photic inputs indicative of arousal and metabolic state from brain stem and thalamic centres: thus, output of the clock returns as input to it, creating a stabilising re-entrant motif that is a common feature at all levels of circadian organisation. The effects of non-photic cues are best exemplified in blind people who are nevertheless able to maintain daily synchrony (Klerman and others 1998), but they are also generally important in situations such as shift-work, in which daily activity, meal and social routines are altered. The synchronised SCN conveys its circadian cues to less powerful clocks distributed across the brain, which in turn control rhythms in their dependent behaviours, including mood and cognition. This co-ordination is mediated by neural efferents, including to the surrounding hypothalamus, the mid-line thalamus and the brain stem (Reppert and Weaver 2002). In parallel, circadian regulation of neuroendocrine and autonomic pathways provides synchronising cues to clocks in peripheral tissues. This ensures adaptive co-ordination of organism-wide physiological and metabolic rhythms that complement and sustain the sleep/ wake and feeding/ fasting cycles. The SCN thereby brings peripheral clocks into step with one another under its consensus internal representation of solar time. With modern lifestyles involving, amongst other challenges, rotational shift work, altered social lives, international air travel and exposure to bright light at night, significant misalignments can occur between internal SCN time and external cues. If the resulting circadian disorganisation is prolonged, it can impose substantial burdens on physiological, metabolic and mental health (Roenneberg and Merrow 2016).
